# Correction: The Screening Visual Complaints questionnaire (SVCq) in people with Parkinson’s disease—Confirmatory factor analysis and advice for its use in clinical practice

**DOI:** 10.1371/journal.pone.0277781

**Published:** 2022-11-07

**Authors:** The PLOS ONE Editors

## Notice of republication

This article was republished on November 4, 2022, to address a file issue. Please download this article again to view the correct version.
