# Correction: The Screening Visual Complaints questionnaire (SVCq) in people with Parkinson’s disease—Confirmatory factor analysis and advice for its use in clinical practice

**DOI:** 10.1371/journal.pone.0278279

**Published:** 2022-11-22

**Authors:** Iris van der Lijn, Gera A. de Haan, Fleur E. van der Feen, Famke Huizinga, Anselm B. M. Fuermaier, Teus van Laar, Joost Heutink

The Data Availability Statement for this article [1] is updated to:

The data that support the findings of this study are available from DataVerseNL. Because of ethical reasons, restrictions apply to the availability of these data (i.e., the combination of variables can potentially lead to participants being identified). Data are available at https://doi.org/10.34894/V9IGKJ with the permission of the ethics board of the Faculty of Behavioral and Social Sciences, University of Groningen (contact: dcc@rug.nl).
